# Draft Genome Sequences of Pseudomonas sp. Strain 382 and Pantoea coffeiphila 342, Endophytic Bacteria Isolated from Brazilian Guarana [Paullinia cupana (Mart.) Ducke]

**DOI:** 10.1128/genomeA.00287-18

**Published:** 2018-04-19

**Authors:** Kátia Aparecida de Siqueira, Rhavena Graziela Liotti, Tiago Antônio de Oliveira Mendes, Marcos Antônio Soares

**Affiliations:** a Department of Botany and Ecology, Laboratory of Biotechnology and Microbial Ecology, Institute of Biosciences, Federal University of Mato Grosso, Cuiaba, Mato Grosso, Brazil; b Department of Biochemistry and Molecular Biology, Federal University of Viçosa, Viçosa, Minas Gerais, Brazil; c Federal Institute of Mato Grosso, Cáceres, Mato Grosso, Brazil

## Abstract

Pseudomonas sp. strain 382 and Pantoea coffeiphila 342 are two endophytic bacterial strains isolated from Paullinia cupana (guarana) seeds. Their draft genome sizes were 5.96 and 6.38 Mbp, with 315 and 266 scaffolds and 52% and 62% GC content, respectively.

## GENOME ANNOUNCEMENT

We have selected two endophytes, Pseudomonas sp. strain 382 and Pantoea coffeiphila 342, of the seeds of Paullinia cupana (1), an Amazonian plant of economic interest.

First, the DNA was extracted using a PowerSoil DNA isolation kit, and the libraries were prepared using a Nextera DNA library preparation kit. After quantification by quantitative PCR (qPCR) and size quality control of libraries using the Agilent 1200 Bioanalyzer (Agilent, USA), the DNA samples were sequenced using the HiSeq 4000 sequencer (Illumina, USA) in the 150-bp paired-end mode. Adapters and low-quality sequences were removed using the software Trimmomatic (2).

The whole-genome sequences of P. coffeiphila 342 and Pseudomonas sp. 382 were *de novo* assembled using SPAdes (3), resulting in 315 and 66 scaffolds, respectively. Their genomes were represented in single linear chromosomes with, respectively, 5,967,657 and 6,385,645 bp, 52% and 62% GC content, and *N*_50_ values of 288,204 bp (mean read length, 555,521 bp) and 178,683 bp (mean read length, 585.472 bp). Genome completeness analysis with BUSCO (4) resulted in 99.5% complete (C) (0.4% duplicated [D]), 0.0% fragmented (F), 0.5% missed (M), and 452 genes (n) for P. coffeiphila 342; and 98% C (2.0% D), 0.2% F, 1.8% M, and 1,452 genes (n) for Pseudomonas sp. 382, indicating that the assembled genome sequences covered most of the coding regions.

Annotation of the genome sequence using the Rapid Annotations using Subsystem Technology (RAST) server (5), followed by tRNA and rRNA detection using ARAGORN (6) and RNAmmer (7), respectively, identified 5,528 coding sequences, 79 tRNAs, and 9 rRNAs in P. coffeiphila 342 and 5,998 coding sequences, 77 tRNAs, and 11 rRNAs in Pseudomonas sp. 382.

### Accession number(s).

The complete genome sequences of Pantoea coffeiphila 342 and Pseudomonas sp. strain 382 were deposited in GenBank under the accession numbers PDET00000000 and PEMX00000000, respectively. The versions described in this paper are the ﬁrst versions.
